# Maintenance Therapy with Immunomodulatory Drugs after Autologous Stem Cell Transplantation in Patients with Multiple Myeloma: A Meta-Analysis of Randomized Controlled Trials

**DOI:** 10.1371/journal.pone.0072635

**Published:** 2013-08-19

**Authors:** Xueshi Ye, Jinwen Huang, Qin Pan, Wanli Li

**Affiliations:** 1 Department of Hematology, Sir Run Run Shaw Hospital, Zhejiang University School of Medicine, Hangzhou, China; 2 Department of Oncology, Sir Run Run Shaw Hospital, Zhejiang University School of Medicine, Hangzhou, China; 3 Department of Orthopedics, the Second Affiliated Hospital, Zhejiang University School of Medicine, Hangzhou, China; Cardiff University, United Kingdom

## Abstract

**Background:**

Although high-dose therapy (HDT) with autologous stem cell transplantation (ASCT) has been confirmed to result in longer remission time than conventional chemotherapy, multiple myeloma (MM) remains incurable. Post-ASCT maintenance is considered as a strategy for obtaining durable remissions and preventing tumor progression. Randomized controlled trials (RCTs) studying maintenance therapy with immunomodulatory drugs (IMiDs) after ASCT have shown some valuable survival improvements. This meta-analysis of RCTs therefore assesses the effect of post-ASCT IMiDs maintenance on MM patients.

**Methods:**

We performed a meta-analysis to evaluate the impact of IMiDs (thalidomide or lenalidomide) as post-ASCT maintenance therapy on the survival of newly diagnosed MM patients. The outcomes for this meta-analysis were progression-free survival (PFS) and overall survival (OS).

**Results:**

Eight RCTs enrolling 3514 patients were included for analysis. An obvious improvement in Os (hazard ratio [HR] 0.75) and a significant PFS advantage (HR 0.58) with post-ASCT IMiDs maintenance was revealed. Thalidomide maintenance after ASCT can result in significant benefit in Os (HR 0.72), particularly combined with corticosteroids (HR 0.66).

**Conclusions:**

MM patients after ASCT have a significant overall survival benefit with IMiDs maintenance. IMiDs maintenance was justified for MM patients who received HDT with ASCT.

## Introduction

Multiple myeloma (MM) is a plasma cell malignancy that comprises about 1% of malignant tumors and 10% to 15% of hematopoietic neoplasms, and causes 20% of deaths from hematologic malignancy [1]. During the past 20 years, high-dose therapy (HDT) followed by autologous stem cell transplantation (ASCT) has become the first line therapy for the eligible younger newly diagnosed patients with MM [2]. Although HDT with ASCT has been confirmed to result in higher response rates and longer remission time than conventional chemotherapy, myeloma recurrence occurs almost universally in patients. To date, MM remains an incurable disease. So post-transplantation maintenance therapy is considered as a strategy for obtaining durable remissions and preventing tumor progression.

An optimal maintenance therapy should prolong progression-free survival (PFS) and furthermore prolong overall survival (OS) with acceptable toxicity. Interferon was the first agent extensively studied as maintenance therapy in MM. Individual trials revealed conflicting results. Of two meta-analyses, one [3] revealed that relapse-free survival and OS were prolonged by 4.4 months and 7.0 months, respectively, following interferon maintenance, and another [4] study showed improved PFS, but a small survival benefit that needed balancing against cost and toxicity. Because of toxic side effects and poor tolerance, maintenance therapy with interferon is rarely used now after transplantation. Corticosteroids have significant activity in MM as a single agent or in combination with other drugs. But some clinical trials [5,6] showed conflicting results with corticosteroid maintenance and cannot supply sufficient evidence to recommend corticosteroids as post-transplantation therapy.

Compared with interferon and corticosteroids, the immunomodulatory drugs (IMiDs) thalidomide and lenalidomide have conferred some improvements, particular in terms of the PFS, when used as maintenance therapy after ASCT. The IMiDs have been the most frequently studied maintenance drugs. Several randomized controlled trials (RCTs) studying post-ASCT thalidomide maintenance have shown a consistent result of prolonged PFS, whereas the benefit in OS was variable. Two RCTs comparing lenalidomide with placebo as maintenance therapy after ASCT have recently been completed [7,8]. These two studies both demonstrated a longer PFS from the time of randomization. However, the impacts of post-ASCT lenalidomide maintenance on OS were different in the two trials. Despite many attempts, the overall efficacy of IMiDs maintenance after ASCT, especially the benefit in OS, has not been adequately evaluated. And the role of post-transplantation maintenance therapy in MM remains controversial. Therefore, we performed this meta-analysis to assess the effect of IMiDs as post-ASCT maintenance therapy on patients with MM.

## Methods

### Data sources and search strategy

We searched for eligible studies in PubMed, Embase (OVID), The Cochrane Library and the Science Citation Index, using the key words “myeloma”, “thalidomide OR lenalidomide” and “maintenance OR consolidation”. The search results were supplemented by manual searches of relevant studies published in the literature or presented at meetings of the American Society of Hematology, American Society of Clinical Oncology, European Hematology Association and International Myeloma Workshop. Additional potentially relevant studies were examined from the reference lists of the trials identified. All the data retrieved were updated to 30 July 2012.

### Study Selection

We reviewed all the titles and abstracts obtained through our search strategy. We reviewed potentially relevant articles in full to ensure that they satisfied the following criteria: (1) study design: RCT; (2) study population: newly diagnosed MM patients treated with induction chemotherapies followed by ASCT; (3) intervention: IMiDs-containing maintenance regimens; (4) control: observation or other non-IMiDs maintenance regimens; (5) outcomes reported: progression-free or event-free survival (PFS/EFS), and OS. Multiple reports of the same study were considered as one study. All potentially relevant articles were reviewed by two independent investigators (Xueshi Ye and Wanli Li).

### Outcome measures

The aim of this meta-analysis is to evaluate the impact of IMiDs as post-ASCT maintenance therapy on the survival of newly diagnosed MM patients. The primary outcome for this review was OS, which was calculated from the date of randomization until death from any cause. Secondary outcome was PFS, which was measured from the date of randomization to the time of disease progression, relapse, or death.

### Study quality assessment

All studies were randomized controlled trials. The methodological quality of the included studies was rated using the Jadad scale, including the reporting of the randomization method, blinding score and completeness of follow-up [9]. The maximal scores for an included study were 5.We arbitrarily classified quality as high (score: 3–5) versus low (score: 0–2).

### Data extraction

Relevant studies were examined through full-text review, and those which met all the inclusion criteria were included in the final analysis. Both investigators independently extracted data (baseline characteristics, outcomes, numbers of events) using a predesigned data extraction form. Any discrepancies between the two investigators at the screening or data extraction stage were resolved by discussion.

### Statistical analysis

Because the outcomes for this review were OS and PFS, the hazard ratio (HR) and 95% confidence interval (95% CI) were chosen to evaluate the effect of IMiDs. In some studies, the data on the HR of OS and PFS could be directly extracted through full-text review. However, other studies used non-HR data on OS and PFS to evaluate the effect of IMiDs. So for these given studies, the HR was estimated using methods described by Tierney et al. [10]. The I^2^ statistic was used to quantify heterogeneity among the studies. Any value of I^2^ less than 25% was considered low heterogeneity, 25% to 50% was moderate heterogeneity, and greater than 50% was defined as high heterogeneity [11]. When the heterogeneity was considered high, a random-effects model was used to pool the HR to assess the impact of IMiDs on OS and PFS. To explore the possible sources of heterogeneity, sensitivity analyses and subgroup analyses were performed. Because of the small number of included studies (eight included trials, of which only seven were published papers), publication bias was not formally assessed. All calculations related to meta-analysis were performed using STATA 11.0.

## Results

### Selection of studies

**Figure 1:**
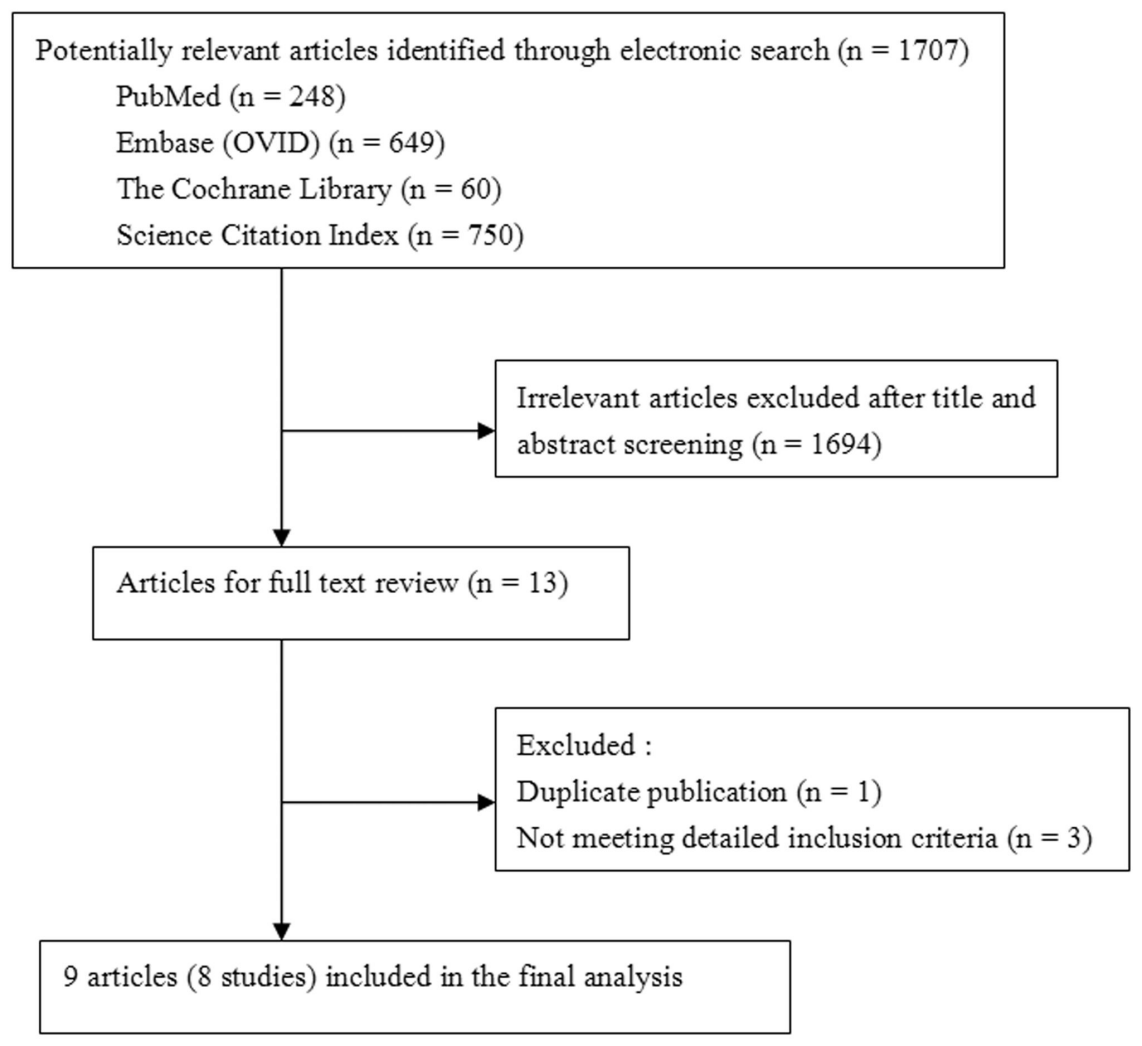
Flow diagram of study selection in the meta-analysis.

We identified 1707 references through a comprehensive search of PubMed, Embase (OVID), The Cochrane Library and the Science Citation Index (Figure 1). Based on title and abstract screening, 14 articles were considered worthy of a thorough evaluation. Following further full-text review of the 14 articles, 1 article [12] was excluded because it reported the same study and duplicate data with 1 included study [13,14]. Three articles [15–17] were excluded because they could not meet the detailed inclusion criteria. In the end, nine articles [7,8,13,14,18–22] meeting all the inclusion criteria were included. Among them, two articles [13,14] that both supplied valuable data from the same study for analysis were considered as one study. So eight RCTs were finally included in this meta-analysis.

The methodological quality of each study is described in Table 1.

**Table 1:** Quality score of studies included.

**Study**	**Randomization**	**Blinding**	**Withdrawal/lost to follow-up**	**Total**
Attal 2006 [18]	2	0	0	2
Barlogie 2006 [13,14]	1	0	1	2
Spencer 2009 [19]	2	0	1	3
Stewart 2010(Abstract)[20]	1	0	0	1
Morgan 2012 [22]	2	0	1	3
Maiolino 2012 [21]	1	0	1	2
McCarthy 2012 [8]	2	0	1	3
Attal 2012 [7]	1	1	1	3

### Description of Trials

In the eight trials, all the patients accepted high-dose chemotherapy followed by ASCT, and then maintenance therapy with or without IMiDs. Seven RCTs were reported as full publications, one [20] only published an abstract. The eight RCTs, whose characteristics are described in Table 2, enrolled 3514 patients considered in this meta-analysis, of which 1643 patients were randomized to the experimental arm with IMiDs as maintenance therapy and 1871 patients to the control arm. Six trials used thalidomide as post-ASCT maintenance. Among the six trials, five trials used thalidomide only as maintenance, while in one study [14] thalidomide was administered during both the induction and maintenance phases. The median duration of thalidomide treatment ranged from 7 to 16 months, and the median follow-up time ranged from 27 to 72 months. In the other two studies [7,8], lenalidomide was used as maintenance therapy after ASCT. The median follow-up time ranged from 34 to 45 months. The MRC myeloma IX study [22] consists of two groups of patients. Before the start of thalidomide maintenance, one group accepted intensive induction therapy (including ASCT), and the other group accepted non-intensive induction therapy. The data on PFS and OS included in this meta-analysis were extracted from the transplant group only.

**Table 2:** Characteristics of the eligible studies.

**Author, year**	**N**	**Median age (y)**	**Maintenance therapy regimen**	**Median duration of IMIDs (m)**	**Median of follow-up time (m)**	**EFS/PFS**	**OS**
Attal, 2006	E: 201	59±8	T + pamidronate	15	39	3-yEFS:52%	4-yOS:87%
	C(A):200	59±8	None		40	3-yEFS:36%	4-yOS:77%
	C(B):196	58±8	Pamidronate		39	3-yEFS:37%	4-yOS:74%
Barlogie, 2006	E: 323	≤ 75	T+ dexamethasone+ interferon	30	72	5-yEFS:56%	5-yOS:67%
	C: 345	≤ 75	Dexamethasone+ interferon			5-yEFS:45%	5-yOS:65%
Spencer, 2009	E: 114	57	T+prednisone	12	36	3-yPFS:42%	3-yOS:86%
	C: 129	57	Prednisone			3-yPFS:23%	3-yOS:75%
Stewart, 2010	E: 166	58	T +prednisone	NA	48	Median PFS:28m	4-yOS:68%
	C: 166	58	None			Median PFS:17m	4-yOS:60%
Morgan, 2012	E: 245	59	T	7	46	Median PFS:30m	3-yOS:75%
	C: 247	59	None			Median PFS:23m	3-yOS:80%
Maiolino,2012	E: 56	52	T+dexamethasone	16	27	2-yPFS:64%	2-yOS:85%
	C: 52	55	Dexamethasone			2-yPFS:30%	2-yOS:70%
Attal, 2012	E: 307	55	Lenalidomide	NA	45	4-yPFS:43%	4-yOS:79%
	C: 307	55	Placebo			4-yPFS:22%	4-yOS:73%
McCarthy, 2012	E: 231	59	Lenalidomide	NA	34	86 events for 34 m	3-yOS:88%
	C: 229	58	Placebo			132 events for 34 m	3-yOS:80%

**Abbreviations: T, Thalidomide; EFS, event-free survival; PFS, progression-free survival; OS, overall survival; E, experimental arm; C, control arm; m: months; y, year; NA, not available.**

### Overall survival

**Figure 2:**
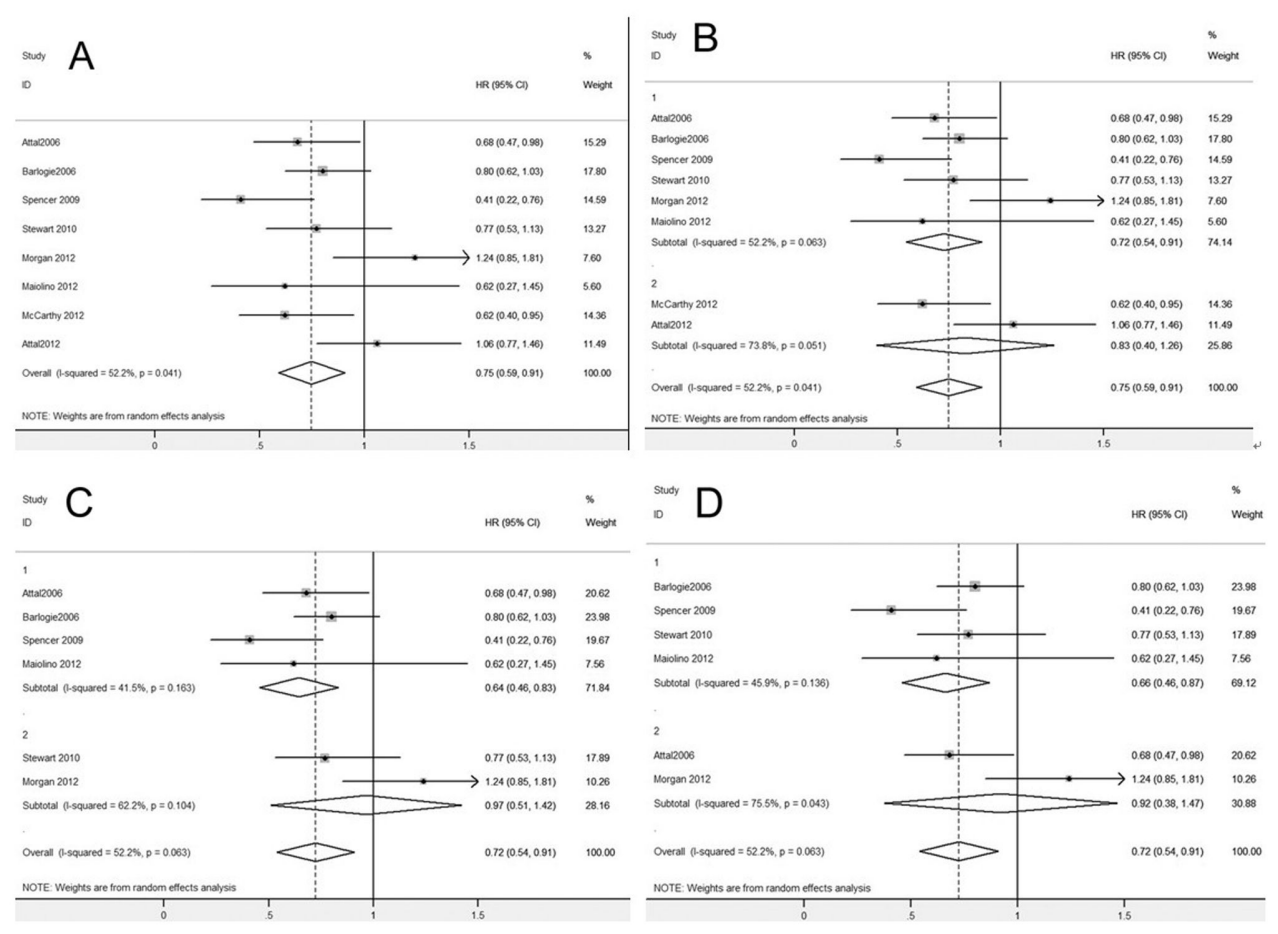
Meta-analysis of overall survival (OS) with IMiDs maintenance after ASCT. (**A**) OS with post-ASCT IMiDs maintenance. (**B**) OS with post-ASCT IMiDs maintenance, subgroup analysis according to thalidomide (Group 1) or lenalidomide (Group 2) as maintenance therapy. (**C**) OS with thalidomide maintenance, subgroup analysis according to non-IMiDs maintenance (Group 1) or no maintenance (Group 2) in the control arm. (**D**) OS with thalidomide maintenance, subgroup analysis according to corticosteroids combined with thalidomide (Group 1) or thalidomide alone (Group 2) as maintenance in the experimental arm. Abbreviations: IMiDs, immunomodulatory drugs.

Figure 2 shows the pooled HR of OS. Our meta-analysis revealed an obvious improvement of OS (HR 0.75, 95% CI: 0.59–0.91, Figure 2A) when post-ASCT IMiDs maintenance was compared with no maintenance or other non-IMiDs maintenance therapies. However, in subgroup analysis (Figure 2B), thalidomide maintenance after ASCT resulted in a significant benefit in OS (HR 0.72, 95% CI: 0.54–0.91), while lenalidomide maintenance after ASCT showed no benefit in OS (HR 0.83, 95% CI: 0.40–1.26). Two other subgroup analyses of the RCTs with thalidomide maintenance after ASCT were performed. Four RCTs in which the control arm involving non-IMiDs maintenance exhibited a significant benefit of OS (HR 0.64, 95% CI: 0.46–0.83, Figure 2C), while the other two trials with no maintenance therapy in the control arm showed no improvement of OS (HR 0.97, 95% CI: 0.51–1.42, Figure 2C). Corticosteroids combined with thalidomide as maintenance therapy after ASCT brought significant benefit in OS (HR 0.66, 95% CI: 0.46–0.87, Figure 2D), while thalidomide as monotherapy showed no benefit in OS (HR 0.92, 95% CI: 0.38–1.47, Figure 2D).

### Progression-free survival

**Figure 3:**
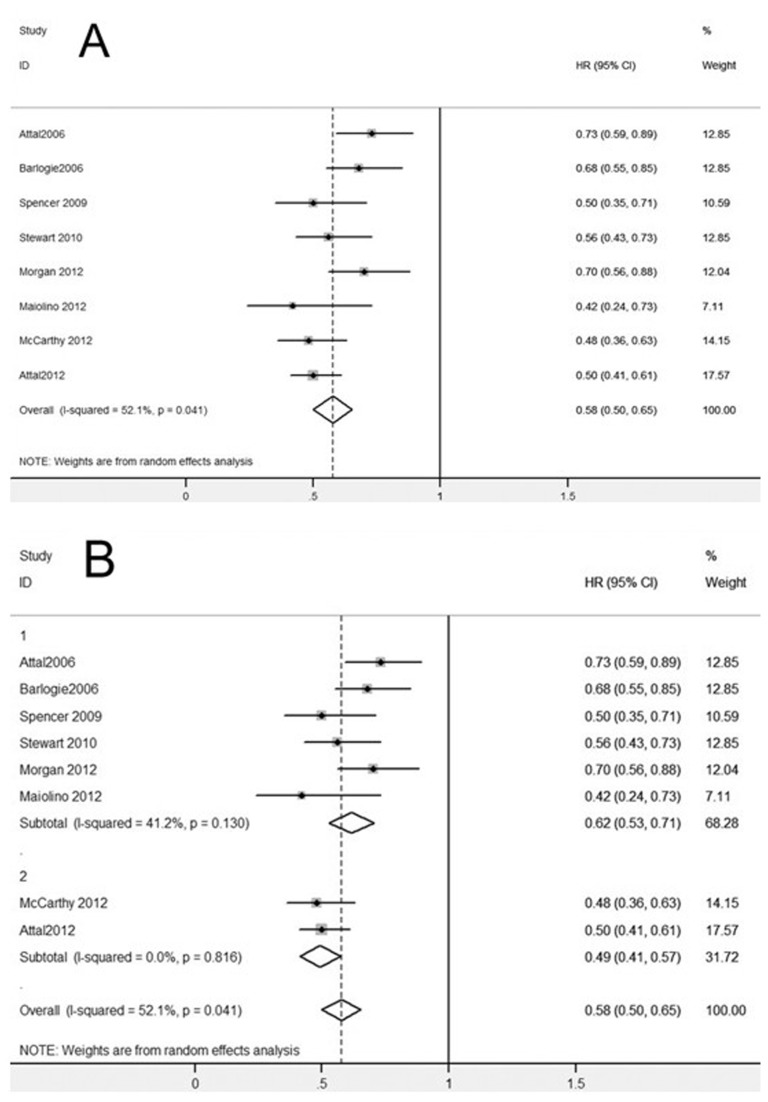
Meta-analysis of progression-free survival (PFS) with IMiDs maintenance after ASCT. (**A**) PFS with post-ASCT IMiDs maintenance. (**B**) PFS with post-ASCT IMiDs maintenance, subgroup analysis according to thalidomide (Group 1) or lenalidomide (Group 2) as maintenance therapy. Abbreviations: IMiDs, immunomodulatory drugs.

Figure 3 shows the results of the meta-analysis of PFS data, demonstrating a significant PFS advantage of post-ASCT IMiDs maintenance (HR 0.58, 95% CI: 0.50–0.65, Figure 3A). And in subgroup analysis (Figure 3B), a significant improvement of PFS was revealed, not only with thalidomide maintenance after ASCT (HR 0.62, 95% CI: 0.53–0.71), but also with lenalidomide maintenance after ASCT (HR 0.49, 95% CI: 0.41–0.57).

### Heterogeneity and sensitivity analysis

There was a statistically significant heterogeneity among all the trials for OS (p = 0.041, I^2^ = 52.2) and PFS (p = 0.041, I^2^ = 52.1). A sensitivity analysis was performed by removing some studies each time and analyzing the heterogeneity across the remaining studies. The results of the sensitivity analysis are shown in Table 3. Exclusion of the Morgan study from the analysis decreased the heterogeneity across the studies for OS and PFS. In addition, removal of the Attal study (2012) and the McCarthy study, which used lenalidomide as maintenance therapy after ASCT, demonstrated reduction in heterogeneity for PFS, but no change for OS. After removal of the Attal study (2006) and the Morgan study, which used thalidomide alone as maintenance, there was a marked reduction in heterogeneity across the rest of the trials with post-ASCT thalidomide maintenance for OS and PFS.

**Table 3:** Sensitivity analyses.

	**I^2^ (%)**		**P**	
**Studies removed**	**OS**	**PFS**	**OS**	**PFS**
Morgan2012	40.8	49	0.119	0.067
McCarthy2012, Attal 2012	52.2	41.2	0.063	0.13
Attal 2006, Morgan2012, McCarthy2012, Attal 2012	45.9	26.9	0.136	0.251

**Abbreviations: PFS, progression-free survival; OS, overall survival.**

## Discussion

High-dose therapy (HDT) with ASCT has been the first-line therapy for eligible younger MM patients during the past 2 decades. Although HDT with ASCT improves complete remission (CR) rates and survival data, almost all patients ultimately relapse. To improve the depth and length of post-transplantation response, maintenance therapy after ASCT has been administered. Several RCTs of IMiDs maintenance after ASCT have shown a consistent benefit of longer PFS. However, the benefit of longer OS is disputed. So it has been unknown whether IMiDs maintenance can improve OS compared with no maintenance or other non-IMiDs maintenance therapy after ASCT. In our meta-analysis, which included eight RCTs and 3514 patients, the answer is yes. Our results demonstrated an obvious improvement of OS (HR 0.75, 95% CI: 0.59–0.91) and a very significant advantage in PFS (HR 0.58, 95% CI: 0.50–0.65) with post-ASCT IMiDs maintenance. Two kinds of IMiDs, thalidomide and lenalidomide, were used as maintenance therapy after ASCT. In subgroup analysis, the improvements of PFS are consistent with both thalidomide and lenalidomide maintenance; however, with post-ASCT thalidomide maintenance, a statistically lengthened OS was shown, while no improvement of OS was shown with post-ASCT lenalidomide maintenance.

A previous meta-analysis performed by Hicks et al. [23] showed a trend of longer OS but no significant improvement (HR 0.61, 95% CI: 0.37–1.01) when thalidomide was given as maintenance therapy following ASCT. Hicks et al. analyzed four trials with post-ASCT thalidomide maintenance, while our meta-analysis excluded one trial not meeting our inclusion criteria [16] and added three new RCTs, thereby yielding a clearer finding: thalidomide maintenance after ASCT can result in significant benefit in OS (HR 0.72, 95% CI: 0.54–0.91).

Recently, another less toxic IMiD, lenalidomide, has been studied. So our meta-analysis also included two new RCTs investigating post-ASCT lenalidomide maintenance [7,8], which were published in the same issue of the New England Journal of Medicine. Although our results have demonstrated that post-ASCT IMiDs maintenance can improve OS, no benefit in OS was shown (HR 0.83, 95% CI: 0.40–1.26) with lenalidomide maintenance in subgroup analysis. The two new trials with post-ASCT lenalidomide maintenance have produced opposite conclusions. The CALGB 100104 study reported significantly increased OS with lenalidomide maintenance [8], whereas the IFM 2005-02 study reported similar OS in both the lenalidomide maintenance arm and the placebo maintenance arm [7]. The different results from the two trials may be due to the differences in the patient population and treatment (induction, the discontinuation of maintenance therapy), or the time of follow-up. Therefore, it is too early to draw a certain conclusion about the effect of post-ASCT lenalidomide maintenance on OS. Longer follow-up and more RCTs are needed to clarify the different findings.

Our study has demonstrated that thalidomide maintenance after ASCT can significantly improve OS. Among the included trials, some used non-IMiDs maintenance in the control arm, while the others used no maintenance therapy. Whether different maintenance therapy used in the control arm could interfere with the conclusions of trials has been unknown. Theoretically, having no therapy in the control arm will increase the opportunity to show the difference between the control and experimental arms. However, in our subgroup analysis, an improved OS (HR 0.64, 95% CI: 0.46–0.83) was shown with non-IMiDs maintenance in the control arm, while no improvement in OS was shown when there was no therapy in the control arm (HR 0.97, 95% CI: 0.51–1.42). These results were inconsistent with the expected outcomes. We hypothesize that one reason for the lack of improvement in OS in patients who did not receive therapy in the control arm, may have been the low (n = 2) number of studies in the reported literature that were included in this subgroup. In addition, the study by Morgan et al. [22] had some degree of heterogeneity, which might have affected the results of the subgroup analysis. As Morgan study was excluded from further analysis, the heterogeneity across studies became insignificant for both PFS and OS (Table 3). The median duration of thalidomide maintenance in the Morgan study was only 7 months, which was much shorter than the other trials, and this was likely to be the main explanation for this heterogeneity. Furthermore, their study showed no improvement in the OS for the patients that received post-ASCT thalidomide maintenance, which may have resulted in part from the short duration of thalidomide maintenance. When we performed the meta-analysis without the Morgan study, the final conclusion that IMiDs significantly improved the OS and PFS did not change. Therefore, we included the Morgan study in our meta-analysis, but we need to take a cautious view about the results of the subgroup analysis. It is true that more RCTs are needed to clarify the results. And we also think that even with non-IMiDs maintenance in the control arm, an improvement in OS has still been shown in the experimental arm, thus making the conclusions of effective post-ASCT thalidomide maintenance more reliable.

Recent studies have demonstrated high response rates of treatment of thalidomide combined with corticosteroids for MM [24,25]. A hypothesized powerful synergy between thalidomide and corticosteroids has been supported by in vitro data [26]. Most studies of combination therapy, however, have focused on it only as an induction therapy. Among the trials included in our study, some used corticosteroids combined with thalidomide as maintenance therapy after ASCT, while the others used thalidomide as monotherapy. Our question is whether corticosteroids combined with thalidomide as maintenance therapy can produce a better effect than thalidomide alone. Our subgroup analysis showed that corticosteroids combined with thalidomide as post-ASCT maintenance brought a significant benefit in OS (HR 0.66, 95% CI: 0.46–0.87), while thalidomide as monotherapy did not (HR 0.92, 95% CI: 0.38–1.47). The analysis implied that corticosteroids combined with thalidomide as post-ASCT maintenance can produce a better treatment response than thalidomide alone. However, because only two studies with thalidomide as monotherapy were included in the analysis, the difference might need more trials to be further confirmed.

Results of the sensitivity analysis revealed statistically significant heterogeneity (test for heterogeneity, p = 0.041, I^2^ = 52.2) for OS and PFS in our analysis. When the Morgan study was excluded from the analysis, the heterogeneity across studies became insignificant for both PFS and OS. The median duration of thalidomide maintenance in the Morgan study being only 7 months, much shorter than in the other trials, may be one of the likely explanations for this heterogeneity. Exclusion of studies with post-ASCT lenalidomide maintenance resulted in disappearance of heterogeneity for PFS, but no change for OS. So this statistical heterogeneity for PFS is likely due to the different IMiDs used in maintenance therapy. When studies with thalidomide alone as maintenance therapy were excluded, the heterogeneity across the rest of the trials with post-ASCT thalidomide maintenance became insignificant in both PFS and OS. Therefore, thalidomide as monotherapy or not was another likely explanation for heterogeneity. In addition, the different patient population, different induction therapy and thalidomide dose may contribute to statistical heterogeneity for PFS and OS.

Cytogenetic abnormalities in multiple myeloma are associated with treatment outcomes. Several trials have tried to identify the differential effects of post-ASCT IMiD maintenance according to different cytogenetic subgroups. The deletion of chromosome 13 is associated with a poor prognosis in MM. The study by Attal et al. in 2006 [18] found that patients without a del13 mutation had a significant benefit from thalidomide, while patients with a del13 mutation did not benefit from thalidomide. In the 2012 from the same group [7], the PFS was shorter in patients with a 13q deletion treated with lenalidomide maintenance therapy. Morgan et al. [22] also showed that the use of thalidomide as a maintenance drug was associated with a PFS benefit and a potential OS benefit in patients with favorable iFISH results, but a worse OS rate was found in patients with an adverse iFISH. However, Barlogie et al. presented an updated analysis [13] that described a significantly better OS rate in the thalidomide arm of high-risk patients as a consequence of the presence of cytogenetic abnormalities defined by conventional karyotyping. The discrepancy between these two studies may have arisen from the different cell characteristics captured by iFISH and conventional karyotyping. These results suggested that patients without FISH-defined cytogenetic risk factors are more likely to benefit from IMiDs maintenance therapy. However, as cytogenetic abnormalities are not sufficient to indicate a poor prognosis due to the various criteria used to subclassify patients, a definitive conclusion remains uncertain.

Recently, the idea of preventing tumor progression and prolonging remission duration with maintenance for myeloma patients has been widely accepted. PFS can be useful as an end point associated with better quality of life. Further, a meaningful OS improvement is necessary to ultimately confirm the efficacy of IMIDs maintenance therapy. Kagoya et al. [27] performed a meta-analysis to compare thalidomide maintenance with other regimens for MM. Their results showed a clear benefit in improved PFS (HR 0.65, 95% CI: 0.59–0.73, p < 0.01) with thalidomide maintenance, but no obvious improvement of OS (HR 0.83, 95% CI: 0.67–1.02, p = 0.07). In our meta-analysis, we evaluated the effect of IMiDs maintenance therapy in specified patient populations that received high-dose therapy followed by ASCT. Our study demonstrated a significant PFS advantage (HR 0.58, 95% CI: 0.50–0.65), and an obvious improvement of OS with IMiDs as maintenance therapy after ASCT (HR 0.75, 95% CI: 0.59–0.91). In subgroup analysis, the same survival benefit was revealed with thalidomide maintenance after ASCT. The difference in OS between the two meta-analyses suggests that those patients who have received HDT with ASCT may be the patient population most likely to benefit from IMiDs maintenance therapy.

There are several limitations of our study. Our meta-analysis used abstracted data, not involving individual patient data. Improved OS with post-ASCT thalidomide maintenance has been confirmed in our study. Lenalidomide, with its better side-effect profile, is considered a very promising maintenance agent for MM. However, no benefit in OS was shown with post-ASCT lenalidomide maintenance in this meta-analysis. This questionable result may be due to only two trials with lenalidomide maintenance being included in the analysis. More RCTs are needed to identify this conclusion. In addition, the different induction therapy and different frequency of transplantation may influence the results in ways our study cannot clarify.

## Conclusions

Our meta-analysis indicates a significant overall survival benefit with IMiDs maintenance for MM patients who received HDT with ASCT. IMiDs maintenance therapy plays an important role in the treatment of MM, and post-ASCT patients may be the population most likely to benefit from it. Thalidomide maintenance after ASCT can result in significant benefit in OS, particularly combined with corticosteroids. However, the effect of post-ASCT lenalidomide maintenance on OS still needs to be clarified by more RCTs with longer follow-up.

## Supporting Information

Checklist S1 **PRISMA Checklist.**
(DOC)
